# Establishment and Characterization of the Novel High-Grade Serous Ovarian Cancer Cell Line OVPA8

**DOI:** 10.3390/ijms19072080

**Published:** 2018-07-17

**Authors:** Patrycja Tudrej, Magdalena Olbryt, Ewa Zembala-Nożyńska, Katarzyna A. Kujawa, Alexander J. Cortez, Anna Fiszer-Kierzkowska, Wojciech Pigłowski, Barbara Nikiel, Magdalena Głowala-Kosińska, Aleksandra Bartkowska-Chrobok, Andrzej Smagur, Wojciech Fidyk, Katarzyna M. Lisowska

**Affiliations:** 1 Center for Translational Research and Molecular Biology of Cancer, Maria Skłodowskaj-Curie Institute—Oncology Center, Gliwice Branch, ul. Wybrzeże Armii Krajowej 15, 44-101 Gliwice, Poland; patrycja.tudrej@io.gliwice.pl (P.T.); magdalena.olbryt@io.gliwice.pl (M.O.); katarzyna.kujawa@io.gliwice.pl (K.A.K.); alexander.cortez@io.gliwice.pl (A.J.C.); 2 Thumor Pathology Department, Maria Skłodowskaj-Curie Institute—Oncology Center, Gliwice Branch, ul. Wybrzeże Armii Krajowej 15, 44-101 Gliwice, Poland; ewa.zembala-nozynska@io.gliwice.pl (E.Z.-N.); barbara.nikiel@io.gliwice.pl (B.N.); 3 Molecular Diagnostics Laboratory, Maria Skłodowskaj-Curie Institute—Oncology Center, Gliwice Branch, ul. Wybrzeże Armii Krajowej 15, 44-101 Gliwice, Poland; anna.fiszer-kierzkowska@io.gliwice.pl (A.F.-K.); wojciech.piglowski@io.gliwice.pl (W.P.); 4 Department of Bone Marrow Transplantation and Hematology-Oncology, Maria Skłodowskaj-Curie Institute—Oncology Center, Gliwice Branch, ul. Wybrzeże Armii Krajowej 15, 44-101 Gliwice, Poland; magdalena.glowala-kosinska@io.gliwice.pl (M.G.-K.); andrzej.smagur@io.gliwice.pl (A.S.); wojciech.fidyk@io.gliwice.pl (W.F.); 5 Department of Hematology and Bone Marrow Transplantation, Andrzej Mielęcki Independent Public Hospital, ul. Dąbrowskiego 25, 40-032 Katowice, Poland; labhem@spskm.katowice.pl

**Keywords:** high-grade serous ovarian cancer, cell line, fibroblast growth factor inhibitor CPL304-110-01

## Abstract

High-grade serous ovarian carcinoma (HGSOC) is the most frequent histological type of ovarian cancer and the one with worst prognosis. Unfortunately, the majority of established ovarian cancer cell lines which are used in the research have unclear histological origin and probably do not represent HGSOC. Thus, new and reliable models of HGSOC are needed. Ascitic fluid from a patient with recurrent HGSOC was used to establish a stable cancer cell line. Cells were characterized by cytogenetic karyotyping and short tandem repeat (STR) profiling. New generation sequencing was applied to test for hot-spot mutations in 50 cancer-associated genes and fluorescence in situ hybridization (FISH) analysis was used to check for *TP53* status. Cells were analyzed for expression of several marker genes/proteins by reverse-transcription polymerase chain reaction (RT-PCR), fluorescence-activated cell sorting (FACS), and immunocytochemistry (ICC). Functional tests were performed to compare OVPA8 cells with five commercially available and frequently used ovarian cancer cell lines: SKOV3, A2780, OVCAR3, ES2, and OAW42. Our newly-established OVPA8 cell line shows morphologic and genetic features consistent with HGSOC, such as epithelial morphology, multiple chromosomal aberrations, *TP53* mutation, *BRCA1* mutation, and loss of one copy of *BRCA2*. The OVPA8 line has a stable STR profile. Cells are positive for EpCAM, CK19, and CD44; they have relatively low plating efficiency/ability to form spheroids, a low migration rate, and intermediate invasiveness in matrigel, as compared to other ovarian cancer lines. OVPA8 is sensitive to paclitaxel and resistant to cisplatin. We also tested two FGFR inhibitors; OVPA8 cells were resistant to AZD4547 (AstraZeneca, London, UK), but sensitive to the new inhibitor CPL304-110-01 (Celon Pharma, Łomianki/Kiełpin, Poland). We have established and characterized a novel cell line, OVPA8, which can be a valuable preclinical model for studies on high-grade serous ovarian cancer.

## 1. Introduction

Preclinical studies in cancer research rely mainly on the established cancer cell lines. Thus, the quality and identity of cell line models is crucial to achieve reliable results. However, several cancer cell lines, which have been in use for tens of years, appear to be misclassified or contaminated. Even within the NCI-60 cell line panel, which is recommended, e.g., for cytotoxicity screening, at least five cell lines were misidentified or misclassified [1]. A well-known example is the MDA-MB-435 cell line which was regarded for many years as a breast cancer line. Later, based on the gene expression profile [2], karyotype analysis, comparative genome hybridization (CGH), and single nucleotide polymorphism (SNP) analysis [3] this line was found to be identical to the M14 melanoma cell line. The discussion concerning the origin of both cell lines (either from breast cancer or from melanoma) is still ongoing [4]. Additionally, the MDA-N cell line, believed to originate from breast cancer, appeared to be identical with MDA-MB-435. Another example is an adriamycin-resistant cell line, MCF-7/ADR-RES, thought to be derived from the breast cancer cell line MCF-7, but later identified as ovarian cancer line OVCAR8 [5]. Other frequently observed phenomenon is cross-contamination of cell lines with the first cell line ever established, namely the HeLa cervical cancer line [6,7]. The aforementioned examples show the importance of the establishment of new, well-characterized cancer cell lines for preclinical studies.

Apart from the problems related with a mix-up of different cell cultures and their cross-contamination, another problematic issue is concerned with their poor original description. Especially, those cell lines that were established many years ago often lack precise information about the patient history, tumor histology, etc. Unfortunately, once in culture, it is difficult to reassess the histological origin of the cells in question.

In the case of ovarian cancer, definite knowledge about the histological origin of its cell lines is particularly vital because the disease is very heterogeneous. In fact, recent studies indicate that “ovarian cancer” is a collective term for several pelvic cancers originating from different tissues and having distinct biology, course of disease, and prognosis [8,9]. Preferably, each histological type of ovarian cancer would require its own dedicated model cell lines. However, many ovarian cancer cell lines have unclear histological origin. Some attempts have been recently undertaken to validate the original histological type of several commercially available cell lines. Particularly comprehensive studies were performed by Domcke et al. and Beaufort et al. [10,11]. Unfortunately, despite thorough morphological and molecular evaluation, including mutation testing and gene expression analysis, the origin of many of these cell lines is still ambiguous.

In western countries, ovarian cancer is a leading cause of death among all gynecological malignancies [9]. The most frequent (about 70% of cases) and with the worst prognosis is high-grade serous ovarian carcinoma (HGSOC). HGSOC is also declared as being the most intensively studied in basic and preclinical research. However, the most frequently used cell lines, e.g., SKOV3 and A2780, probably do not represent this type of ovarian cancer.

Here, we present a new cell line, OVPA8, which is derived from a patient with histologically-confirmed HGSOC. The results of our detailed in vitro analyses indicate that the OVPA8 cells have morphologic, molecular, and functional features of HGSOC. Some analyses were carried out in comparison to five other ovarian cancer cell lines: SKOV3, OVCAR3, OAW42, ES2, and A2780. The OVCAR3 cell line has been used as the HGSOC control, and OAW42 is most probably derived from serous ovarian cancer, while the three others, although very popular, have unclear histological origin, which is still a matter of debate.

## 2. Results

### 2.1. Patient History

**Figure 1:**
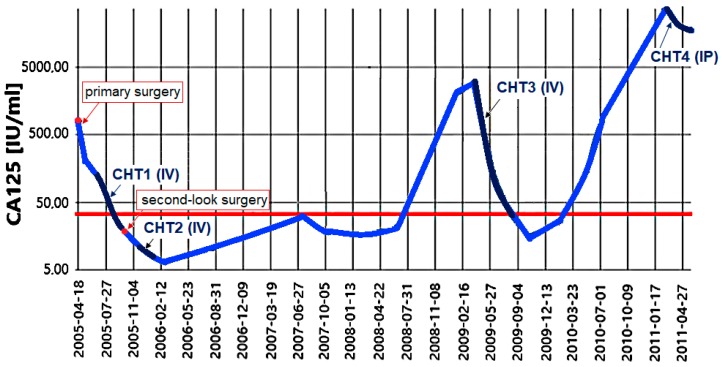
Changes in serum concentration of cancer antigen 125 (CA125) during the course of disease in an OVPA8 patient (logarithmic scale). The CA125 normal value is <35 IU/mL (the threshold value marked by the red line). CHT1—first line chemotherapy (cisplatin and paclitaxel), CHT2—carboplatin and cyclophosphamide, CHT3—carboplatin and paclitaxel, CHT4—cisplatin, IV—intravenous treatment, IP—intraperitoneal treatment.

A 41-year old Caucasian woman was diagnosed with ovarian cancer (papillary serous adenocarcinoma, G3, FIGO IIIC) in 2005. After non-optimal surgical debulking, encompassing bilateral adnexectomy, appendectomy, and omentectomy, she was treated with six cycles of cisplatin and paclitaxel. Then, second-look surgery was performed (hystecectomy, together with pelvic and para-aortic lymphadenectomy). Since multiple focal neoplastic lesions were found in the rectovaginal pouch, she was treated with an additional three cycles of cyclophosphamide and paclitaxel. In January 2009, the CA125 level raised to 2027.6 IU/mL (Figure 1) and multifocal recurrence was confirmed by computed tomography (CT) scan. She was treated with carboplatin and paclitaxel and achieved partial response, however, in 2010 a CT scan revealed recurrent disease. In 2011 she was qualified for the treatment for malignant ascites with intraperitoneal (IP) chemotherapy. Ten liters of ascitic fluid were drained out on 23 February 2011, part of which was used for the establishment of the ovarian cancer cell line. IP chemotherapy with carboplatin was administered to the patient, followed by two cycles of intravenous carboplatin (monotherapy). Before a third cycle she developed massive pleural effusion which was managed by talc pleurodesis. Subsequently, she was qualified for palliative chemotherapy with doxorubicin and received two cycles, the last one at 15 June in 2011. Then, the patient was lost from follow-up.

### 2.2. Establishment of Cell Line Form a High-Grade Serous Ovarian Cancer

**Figure 2:**
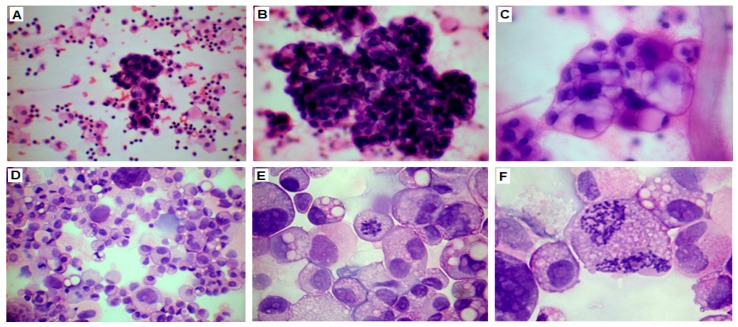
HE staining of ascitic fluid preparations. (**A**,**D**) Cellular fraction of ascitic fluid was heterogenous. (**A**) Tumor cells forming characteristic pseudopapillary structures are visible in the center, multiple red blood cells and leukocytes are visible throughout, and other non-cancerous cells correspond to fibroblasts and mesothelial cells; (**B**,**C**) Cancer cells, with polymorphous, hyperchromatic nuclei forming papillary structures; (**E**,**F**) Pathological mitoses and numerous nucleoli (magnification: 40× (**A**); 100× (**B**,**D**); 200× (**C**); 400× (**E**,**F**)).

The ascitic fluid samples were cytospined, stained with hematoxylin and eosin (HE), and evaluated in the light microscope (Figure 2). The fluid had abundant tumor cells with characteristically large, polymorphous nuclei. Cancer cells formed characteristic pseudopapillary structures. There were also many red blood cells, leukocytes, and other non-cancerous cells which could correspond to fibroblasts and mesothelial cells exfoliated from the peritoneum.

**Figure 3:**
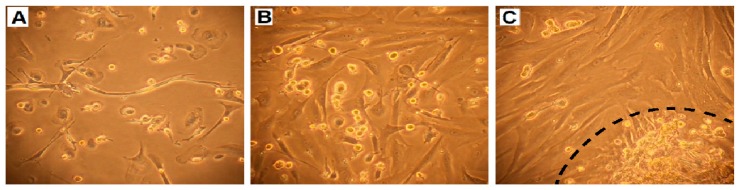
Initially, cell culture from ascitic fluid was heterogeneous and consisted of cancer cells, fibroblasts, and mesothelial cells (**A**–**B**). In higher confluency cancer cells tended to grow as spheres (**C**—inside the dotted line) on the layer of non-cancerous cells (Axiovert 40 CFL Inverted Phase Contrast Microscope, Zeiss, Oberkochen, Germany; magnification: 100×).

One liter of the ascitic fluid was used for separation and culturing of cancer cells, according to Langdon et al. [12]. First, the red blood cells were eliminated by centrifugation with Histopaque. To imitate the in vivo environment, the early passages were carried out using autologous ascitic filtrate, instead of bovine fetal serum. At the initial stage, a large proportion of the cell culture consisted of fibroblasts and mesothelial cells (Figure 3), but they were gradually eliminated by selective trypsinization and multiple passaging.

By now, the cells have been passaged 69 times and the cell culture is morphologically homogenous. Short tandem repeat (STR) profiling was performed at the 36th and 59th passages, showing unique and stable identity of the OVPA8 cell line (Table 1 and Figure S1).

### 2.3. Cell Growth, Morphology and Cytogenetic Characteristics

**Figure 4:**
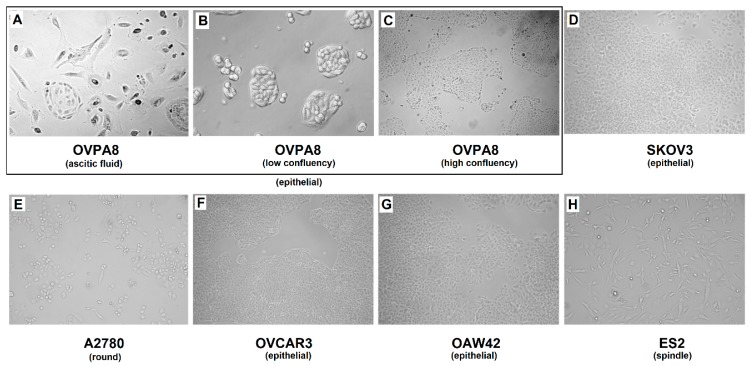
Cellular morphology of ovarian cancer cell lines (phase-contrast microscopy). OVPA8 (**A**–**C**), SKOV3 (**D**), OVCAR3 (**F**), and OVA42 (**G**) cells showed epithelial morphology. A2780 cells (**E**) have round-shaped morphology (according to [10]), while ES2 cells (**H**) have spindle-shaped morphology [10]. The OVPA8 cells show tendency to grow in groups both in early culture of ascitic fluid (**A**) and in established cell line, especially at low confluency (**B**), when they form characteristic islets (Axiovert 40 CFL Inverted Phase Contrast Microscope, Zeiss; magnification 100×).

Starting from early passages, the OVPA8 cells have shown relatively low morphological heterogeneity. The cells are medium sized, with epithelial morphology, and indistinct cell borders (Figure 4A–C). The nuclei are centrally located, round, vesicular, and contain visible nucleoli. Cells show adherent growth and have a doubling time of about 44 h. For comparison we used five commercially available ovarian cancer cell lines: SKOV3, A2780, OVCAR3, OAW42, and ES2. Their doubling times show great diversity: for A2780 it is about 18 h; for ES2 it is about 19 h; for SKOV3 it is about 27 h; for OAW42 it is about 34 h; and for OVCAR3 it is about 50 h. We also evaluated the cellular morphology of these cell lines (Figure 4D–H) and the results were concordant with those described in [10] (For summary see Figure 14).

Being resistant to the sub-optimal culture conditions (e.g., old medium, high confluence), OVPA8 cells are easy to maintain. They prefer to grow in close contact, within groups/islets (Figure 4A–C), thus, it is recommended to seed approx. 1 × 10^5^ cells/cm^2^ to avoid long-lasting lag phase after subculturing. When the confluence reaches 100%, the cells start to grow closer, but still in a monolayer, and without evidence of massive cell death, which means that they are resistant to anoikis. Under optimal culture conditions they form almost no floating particles (culture medium looks clear). They tend to adhere strongly to the culture plate surface and prolonged incubation (8–10 min) with trypsin is required for passage. Thus, they may not be a suitable model for the studies of cell surface proteins, which may be damaged by prolonged trypsinization. Despite high adhesiveness to the tissue culture plate surface, they do not form aggregates after detachment from the plate.

**Figure 5:**
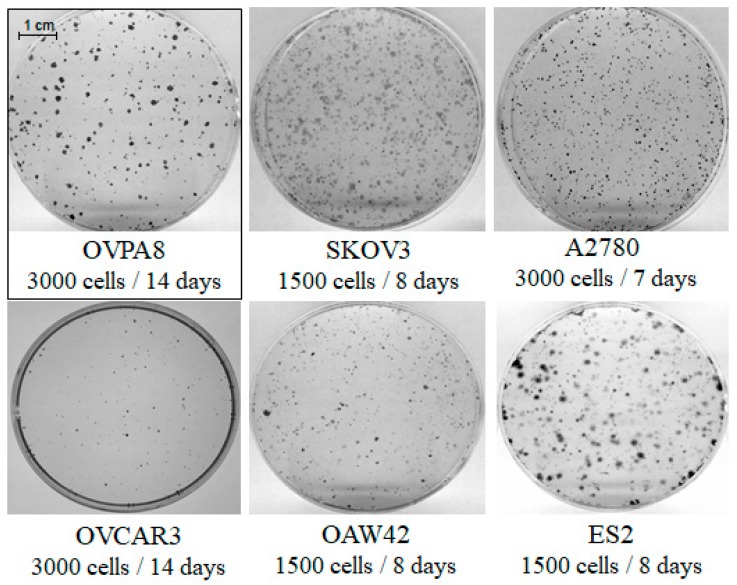
Clonogenic growth of OVPA8 and other ovarian cancer cells. Cells were seeded at the indicated densities and grown for 8–14 days. Afterwards, colonies were fixed, stained with crystal violet, and imaged. The picture shows representative digital images of colonies from one out of three technical replicates from 2–3 independent experiments.

We compared the clonogenicity of OVPA8 cells against five other ovarian cancer cell lines. The OVPA8 cells showed low clonogenic potential in comparison to SKOV3, A2780, OAW42, and ES2 cells. On the contrary, they had much higher plating efficiency than OVCAR3 cells, which, in the same conditions, gave rise only to solitary clones (Figure 5).

**Figure 6:**
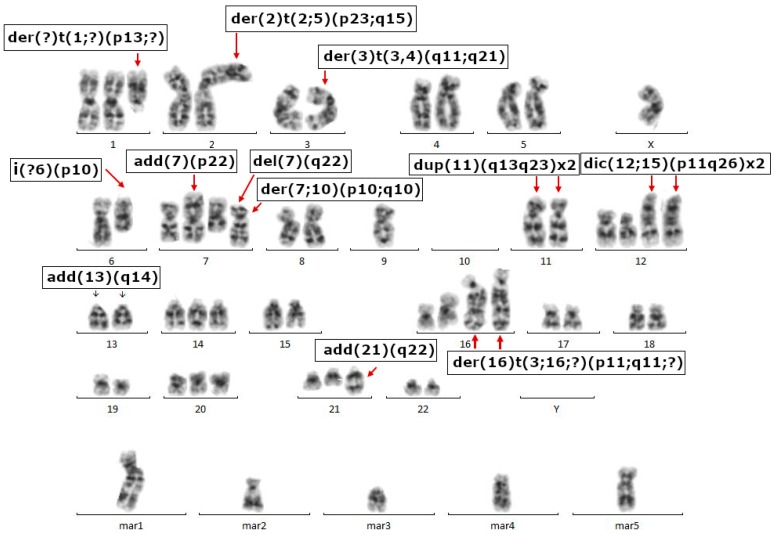
An example of the OVPA8 karyotype with the main chromosomal aberrations indicated.

Cytogenetic analysis was performed at the 59th passage on seven G-banded metaphase spreads. The pictures were consistent with HGSOC characteristics: we observed abnormal chromosome counts, ranging from 57 to 67, and a very large number of structural aberrations, including: der(2)t(2;5)(p23;q15), add(7)(p22), del(7)(q22), dup(11)(q13q23)x2, dic(12;15)(p11;q26)x2, der(?)t(1;?)(p13;?), add(21)(q22), der(16)t(3;16;?)(p11;q11;?)x2, der(3)t(3;4)(q11;q21), add(13)(q14), i(?6)(p10), der(6;10)(p10;q10), dup(11)(q12q23), der(7;12)(p10;q10), der(7;10)(p10;q10), and from five to nine marker chromosomes. An example of the karyotype is shown in Figure 6.

### 2.4. Microbiological Analysis and Biosafety

**Figure 7:**
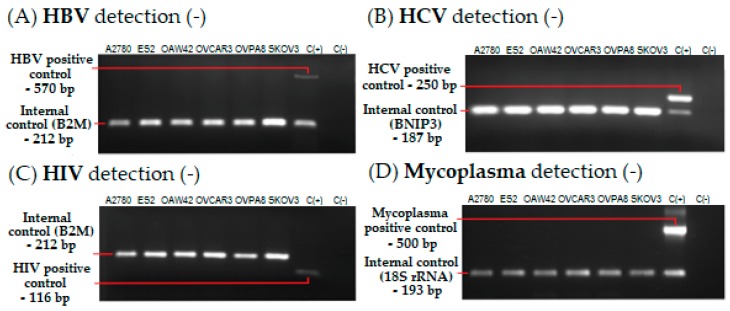
PCR-based detection of HBV (**A**), HCV (**B**), HIV (**C**), and mycoplasma (**D**) infection. Each cell line was found to be free from the viruses. Mycoplasma testing was performed regularly for all cell lines used in the experiments; C(+)—positive control, C(−)—negative control.

To evaluate the biosafety of OVPA8 line we performed PCR reactions designed to detect the following pathogenic viruses: EBV, HBV, HCV, and HIV. We also tested five commercial ovarian cancer cell lines. Of the latter, three lines sold by ATCC (SKOV3, OVCAR3, and ES2) are assigned to the biosafety level 1 (BSL1), although the producer states that they were not tested for the presence of viruses. A2780 and OAW42 which are sold by ECACC are assigned to BSL2 (also not tested for viral contamination). We found that OVPA8 cell line was free from these viruses, thus working with this line does not require elevated biosafety measures. SKOV3, OVCAR3, OAW42, ES2, and A2780 were also proved to be virus-negative (Figure 7A–C). In addition, each cell line was PCR tested for mycoplasma infection and they were found negative throughout all experiments (Figure 7D).

### 2.5. Mutation Profile of OVPA8 Cell Line

**Figure 8:**
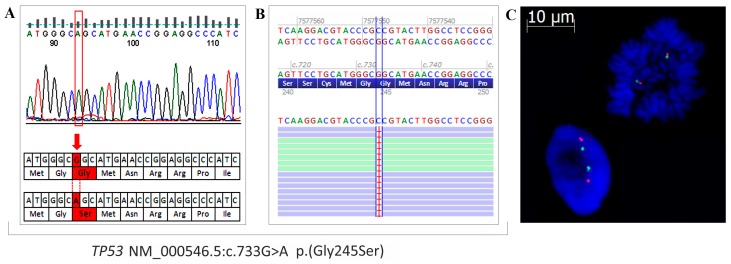
Detection of *TP53* mutation in OVPA8 cell line. Sanger sequencing showed homozygous mutation c.733G>A (p.Gly245Ser in OVPA8 cells (**A**). NGS analysis confirmed this result (**B**), FISH analysis showed no numerical changes in chromosome 17 and *TP53* signals. Green dots represent the chromosome 17 centromere, while orange dots represent *TP53* (**C**)*.*

The frequency of *TP53* mutations in HGSOC is estimated at 96%. The most frequently mutated hot-spots of *TP53* are localized in exons 5–8 [13]. We analyzed OVPA8 and five other ovarian cancer lines by Sanger sequencing of these exons. We found homozygous mutation c.733G>A (p.Gly245Ser) in OVPA8 cell line (Figure 8A) which has been described as pathogenic in VarSome genomic variation databases and ‘likely pathogenic’ in ClinVar database [14]. The results were additionally confirmed by NGS analysis (Figure 8B). Using FISH analysis we also checked whether homozygous reading of this mutation in a Sanger sequencing plot could be related with a loss of a second copy of *TP53*. The results showed no numerical changes in chromosome 17 and *TP53* itself in OVPA8 cells (Figure 8C).

We also confirmed the presence of previously-reported *TP53* mutations in three other cell lines: ES2 (c.722C>T, p.Ser241Phe) [15], OVCAR3 (c.743G>A, p.Arg248Gln) [16], and SKOV3 (c.267delC, p.Ser90Profs) [16]. Additionally, in the OAW42 cell line, we found a c.639A>G (p.Arg213=) polymorphism which has been previously observed in breast cancer [17].

**Figure 9:**
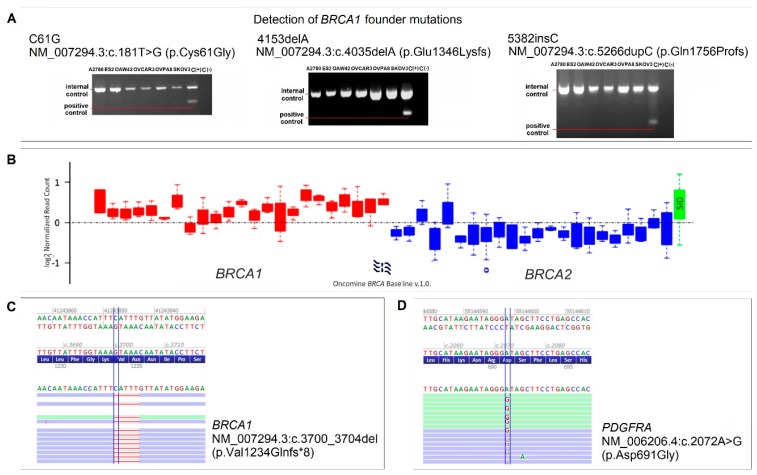
Detection of *BRCA1/2* and *PDGFRA* mutations. The ASO-PCR analysis of three founder mutations of *BRCA1* gave negative results in all cell lines, including OVPA8 (**A**); Loss of one copy of BRCA2 was detected using NGS data for OVPA8 cells. The graph shows the visualization of the distribution of normalized amplicon coverage values across BRCA1 and BRCA2 coding exons and control amplicons (SID) (**B**); NGS detected homozygous pathogenic mutation in *BRCA1* (**C**) and a heterozygous variant of unknown meaning in *PDGFRA* (**D**); C(+)—positive control, C(−)—negative control.

High-grade serous ovarian cancer frequently presents dysfunction of *BRCA* genes (either germline or somatic mutations, promoter methylation, and/or loss of heterozygosity). We checked OVPA8 cells for three *BRCA1* founder mutations which are most common in Polish population: C61G (c.181T>G; p.Cys61Gly), 4153delA (c.4035delA; p.Glu1346Lysfs), and 5382insC (c.5266dupC; p.Gln1756Profs). The analysis gave negative results (Figure 9A). Subsequently, we analyzed *BRCA1* and *BRCA2* by NGS using Oncomine BRCA Research Assay, and we found that OVPA8 cells contain homozygous pathogenic mutation c.3700_3704delGTAAA (p.Val1234Glnf) in *BRCA1* (Figure 9C). In addition, we found the loss of one copy of *BRCA2* (Figure 9B), which is consistent with the loss of one whole chromosome 13 visible in three out of seven analyzed karyotypes.

Interestingly, *BRCA1* and *TP53* are both localized on chromosome 17, and both carry homozygous mutations. Chromosome 17 was shown diploid in FISH and in cytogenetic analysis, which may suggest nondisjunction and reduplication of this chromosome.

We further analyzed genetic profile of OVPA8 cells using NGS and the Ion AmpliSeq™Cancer Hotspot Panel v2 which allows detection of approximately 2800 COSMIC mutations within 50 oncogenes and tumor suppressor genes. NGS confirmed the presence of c.733G>A (p.Gly245Ser) mutation in *TP53* gene in OVPA8 cells, and excluded mutations typical for low-grade serous ovarian cancers, i.e., in the *KRAS*, *BRAF*, and *ERBB2* genes. In addition, heterozygous variant (p.Asp691Gly) of unknown meaning was detected in *PDGFRA* gene (Figure 9D). Lack of mutations in large panel of cancer related genes is consistent with HGSOC characteristics, as this histological type of ovarian cancer has low mutational load, except TP53 and BRCA1/2.

### 2.6. Migration and Invasiveness of OVPA8 Cells

**Figure 10:**
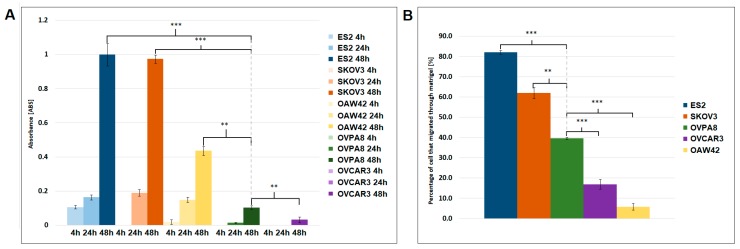
Analysis of cell migration and invasiveness. (**A**) Transwell cell migration assay. The OVPA8 cells showed low migration rate comparing to other ovarian cancer cell lines, except OVCAR3. Statistical significance is indicated for the results obtained after 48 h. For other time points similar significance level was obtained; (**B**) Matrigel invasion assay (96 h). The OVPA8 cell line showed intermediate invasion ability comparing to other ovarian cancer cell lines; **—*p* < 0.001 ***—*p* < 0.0001.

The ability of cancer cells to migrate and invade the basement membrane and extracellular matrix is an important, metastasis-promoting feature. We used a transwell cell migration assay and a matrigel invasion assay to test OVPA8 cells in comparison to other ovarian cancer lines. ES2 and SKOV3 showed the highest migration rate, OAW42 had intermediate migration rate, while OVPA8 was migrating very slowly, similarly like OVCAR3 cells (Figure 10A). In the matrigel assay, ES2 and SKOV3 cells showed the highest invasiveness, while OVCAR3 and OAW42 showed the lowest. The OVPA8 cells had the intermediate invasiveness in comparison to the other cell lines tested (Figure 10B).

### 2.7. Immunophenotypic Characterization of Ovarian Cancer Cell Lines

Immunophenotypic characterization was performed on cytospin preparations of OVPA8 and other cell lines; early and late passages of OVPA8 were used. Using specific primary antibodies we checked expression of the following proteins: Wilms tumor 1 protein (WT1), paired box 8 (PAX8), calretinin, CD68, CD44, luminal cytokeratin 19 (CK19), and Epithelial Cell Adhesion Molecule (EpCAM).

**Figure 11:**
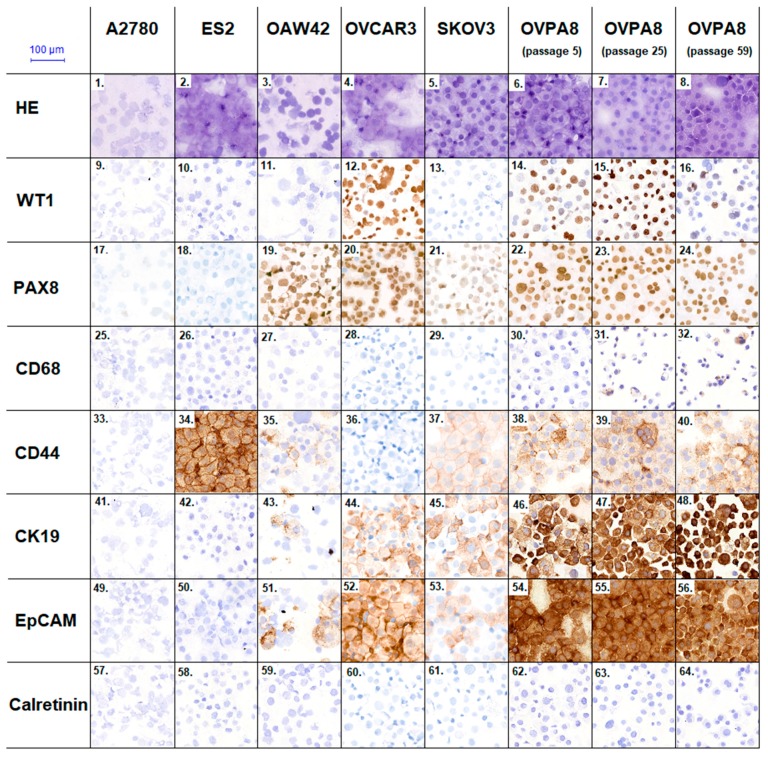
Immunophenotypic characterization of OVPA8 and other ovarian cancer cell lines (magnification: 200×). HE—hematoxylin-eosin staining; WT1—Wilms tumor 1 protein; PAX8—paired box 8 protein; CK19—luminal cytokeratin 19; EpCAM—epithelial cell adhesion molecule.

WT1 protein is proposed as a nuclear marker for serous ovarian cancer [18,19]. We detected the expression of WT1 in a majority of the cells from the OVPA8 cell line (Figure 11(14–16)). Interestingly, stronger staining for this marker was detected in the subpopulation of OVPA8 cells characterized by small nuclei and forming cell clusters. Strong and ubiquitous expression of WT1 was also detected in OVCAR3 cell line (Figure 11(12)), while in A2780 line it was present only in the single cells (Figure 11(9)). We did not observe WT1 expression in SKOV3, OAW42, ES2, and A2780 cells.

Expression of CD68 was checked to detect contamination of the cell culture with macrophages. As expected, in commercially available cell lines there was no CD68 expression, however, in the OVPA8 line single CD68-positive cells were observed (Figure 11(30–32)).

Currently, it is proposed that ovarian tumors with a müllerian phenotype (serous, endometrioid, and clear cell) are not transdifferentiated from mesothelium, as previously considered, but are derived directly from müllerian-type tissues. It has been demonstrated that the majority of HGSOC originate from fallopian tubal secretory epithelial cells, which are positive for the PAX8 transcription factor involved in müllerian cell differentiation [18,20,21]. The OVPA8 cells showed strong, ubiquitous nuclear staining with anti-PAX8 Ab in all three passages analyzed (Figure 11(22–24)), which indicates the müllerian origin of these cells. A similar pattern of PAX8 staining was also observed in OVCAR3 and OAW42 cells. Weaker PAX8 expression was found in SKOV3 cells, while no expression was detected in ES2 and A2780. The latter finding was surprising, but similar observations were also made by others (no PAX8 expression in ES2 and weak expression in A2780) [22].

Calretinin, which is a marker of mesothelial cells, is expressed in ovarian surface epithelium, while not in cancer cells [20]. We expected contamination with mesothelial cells at the early passages of the OVPA8 line. However, no calretinin-expressing cells were observed, neither in the early, nor late, passages of OVPA8 (Figure 11(62–64)). No calretinin expression was either observed in the other established ovarian cancer cell lines (Figure 11(57–61)), which seems to confirm their müllerian origin.

We also tested for the epithelial markers EpCAM and CK19. In [10], the expression of these markers was significantly associated with epithelial morphology of ovarian cancer cells and with serous histology of the source tumor from which the cell line was derived. Both proteins are also proposed as negative prognostic markers in different cancers [23]. The OVPA8 cells showed very high and ubiquitous expression of both markers (Figure 11(46–48, 54–56)). The results for other cell lines were concordant with those shown in [10], except for OVCAR3 line which was shown EpCAM-negative in [10] but in our experiment it displayed moderately intensive and ubiquitous expression of EpCAM (Figure 11(52)).

### 2.8. Evaluation of Cancer Stem-Like Cell Markers

**Figure 12:**
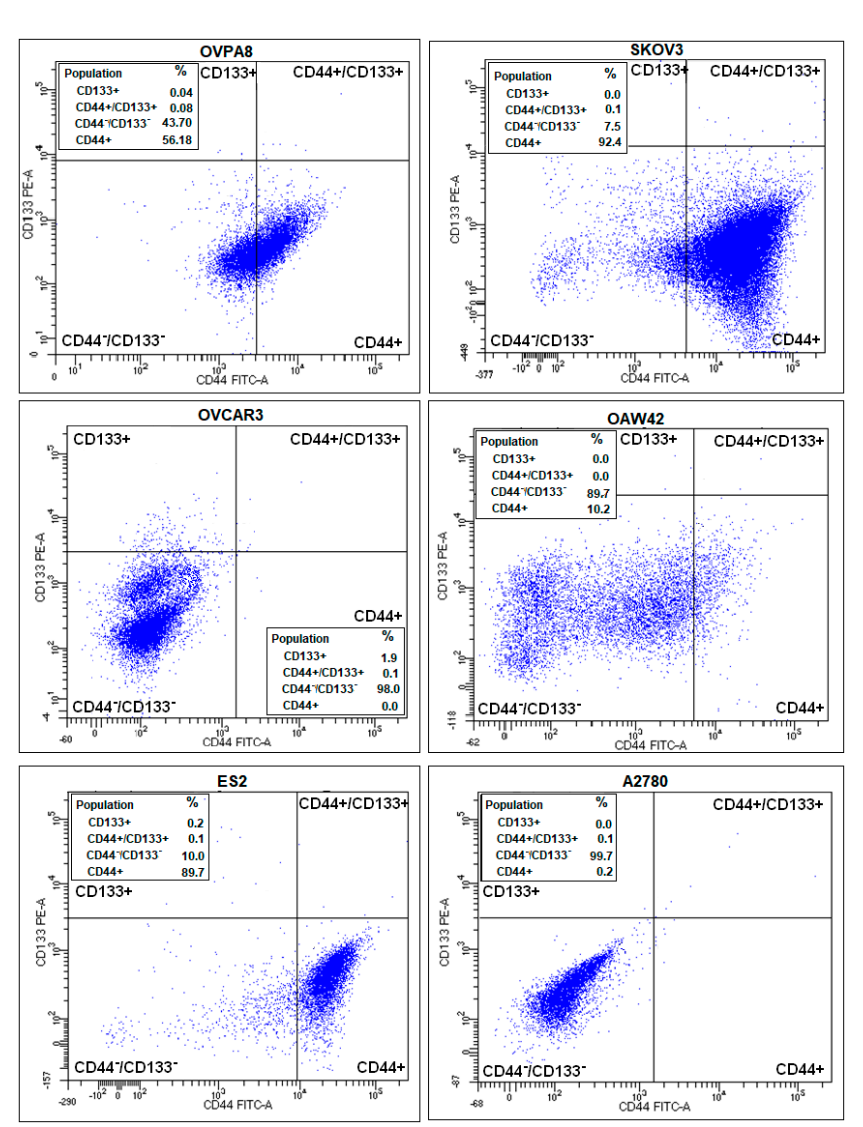
Detection of putative cancer stem-like cells (CSLC) in OVPA8 and other ovarian cancer cell lines. Double staining and flow cytometry detection of CD44 and CD133 markers was performed.

So far, no universal marker combination has been found to faithfully indicate ovarian cancer stem-like cells (CSLC), although surface proteins CD44 and CD133 are proposed by several authors (reviewed in: [24,25]). Flow cytometry (FC) detection of CD44 and CD133 showed that the majority of ovarian cancer lines tested contained approximately 0.1% of CD44+/CD133+ cells (putative CSLC). Only in OAW42 were we not able to detect such cells (Figure 12), possibly due to their low count (below detection threshold).

In general, CD133+ cells were very rare in ovarian cancer cell populations (up to 2% in OVCAR3 and ES2 line). On the contrary, CD44+ cells were much more ubiquitous, reaching about 56% in OVPA8, 89% in ES2 and 92% in SKOV3 line (Figure 11). CD44 is a cell-surface glycoprotein involved e.g., in cell-cell interactions and adhesion. In ovarian cancer, increased density of CD44-positive cells was shown to be associated with chemotherapy resistance [26,27]. High expression of CD44 observed in OVPA8 cells is consistent with their biological characteristics (in vitro cisplatin resistance, see below) and patient history (chemo-resistant relapse). The FC results obtained for CD44 were concordant with those found by immunocytochemistry (ICC) (Figure 11).

As to the other cell lines, we observed strong expression of CD44 in ES2, weak expression in OAW42 and SKOV3, and no expression in A2780 and OVCAR3. For ES2 and A2780 our results were consistent with those described in [10], while for three other cell lines the results were inconsistent.

FC was also applied to detect side population (SP), a small subset of cells capable of rapid removal of cytotoxic compounds. SP is proposed as an equivalent of CSLCs, although, this is not fully confirmed, e.g., in glioblastoma multiforme, side population was found neither sufficient nor necessary for a CSLC phenotype [28]. In our study, SP was detected only in ES2 cell line (0.2% of cells; not shown), and did not correspond to the CD133+/CD44+ population in ovarian cancer cell lines.

### 2.9. Response to Anticancer Drugs

To evaluate drug dose response of the OVPA8 cells, in comparison to other ovarian cancer cell lines, cytotoxicity assays were performed and the half maximal inhibitory concentrations (IC50) were determined for cisplatin, paclitaxel, and for the novel inhibitor of the fibroblast growth factor receptor (FGFR), CPL304-110-01 (CelonPharma) [29], and the control FGFR inhibitor, AZD454 (AstraZeneca) (Table 2). OVPA8 cells were resistant to cisplatin, which is consistent with the patient history (the cell line was derived from recurrent, platinum-resistant disease). They were, however, sensitive to paclitaxel (IC50 = 0.001 µM). Other ovarian cancer cell lines showed low sensitivity to cisplatin and were all highly sensitive to paclitaxel. These results are roughly similar to those shown in [10].

In addition, we found that OVPA8 cells were resistant to AZD4547, while they showed low sensitivity to the novel inhibitor CPL304-110-01 (Table 2, Figures S2–S4). A similar trend, i.e., greater sensitivity toward CPL304-110-01 in comparison to the control AZD4547 inhibitor was also observed in other ovarian cancer cell lines, except for OVCAR3 cells, which were resistant to both inhibitors.

### 2.10. Expression of Potential Prognostic Markers in OVPA8 Cell Line

In our previous study, using expression microarrays we identified a multigene signature related with worse prognosis in HGSOC. We observed that patients with higher expression of these genes in the tumors had shorter overall survival (OS) than patients with lower expression [30]. We further confirmed by immunohistochemistry that higher expression of two genes from this signature, coding for fibronectin (*FN1*) and periostin (*POSTN*), was related with shorter OS (unpublished). Here, we checked the expression level of these two potential prognostic markers in OVPA8 and in other ovarian cancer cell lines, using semi-quantitative RT-PCR and Western blotting methods.

**Figure 13:**
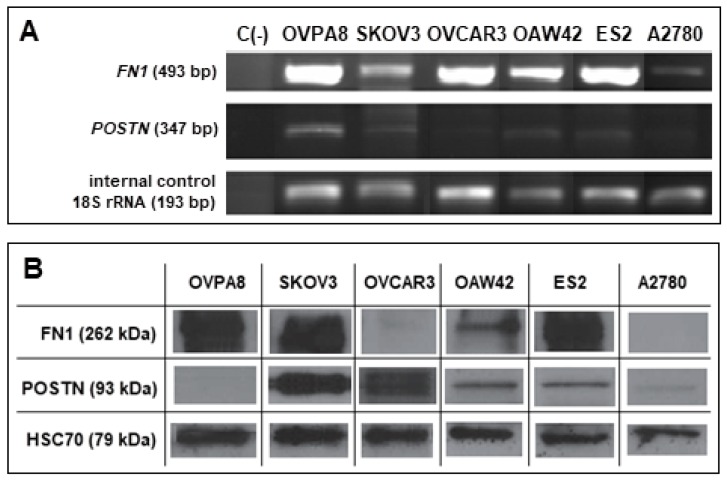
Detection of POSTN and FN1 in OVPA8 and other ovarian cancer cell lines. Gene expression was tested on mRNA level by semi-quantitative RT-PCR (**A**) and at protein level by Western blotting (**B**); C(−)—negative control.

Weak expression of *POSTN* mRNA was observed in all six cell lines (Figure 13A). The highest level of periostin mRNA was observed in OVPA8 cells, while the lowest was in A2780 and OVCAR3. There was no direct correlation between *POSTN* mRNA and protein level. The highest amount of POSTN protein was observed in SKOV3 cell line, while in OVPA8 the lowest (Figure 13B). These differences may be related to variable stability of mRNA and/or protein in different cell lines. 

*FN1* mRNA was expressed in all six cell lines, with the highest level in OVPA8, ES2, and OVCAR3, moderate in OAW42, and low in SKOV3 and A2780 (Figure 13A). At a protein level the highest expression of fibronectin was observed in ES2, OVPA8, and SKOV3, while we did not observe the presence of FN1 in A2780 and OVCAR3 cell lines (Figure 13B).

Further studies are necessary to elucidate the discrepancy between mRNA and the protein level of both genes.

## 3. Discussion

### 3.1. Ovarian Cancer Is a Heterogeneous Disease

Ovarian cancer has always been recognized as a heterogeneous disease with main histological types like serous, endometrioid, clear cell, and mucinous. At first, it was thought that all of these cancers initiate in ovarian surface epithelium (OSE), which is of mesothelial origin. However, the wider the spectrum of immunophenotypic, genetic, and molecular markers available, the greater the differences observed between these histological types themselves, and between them and OSE. For example, it was found that the majority (although not all) of serous ovarian cancers show common features with fallopian tube epithelium, while endometrioid and clear cell cancers show features with the endometrium, the tissues which both have müllerian origin. This phenomenon was initially interpreted as a result of tissue transdifferentiation during carcinogenesis. However, accumulating evidence suggest that these cancers do not undergo transdifferentiation, but rather originate directly from different müllerian epithelia. It is now widely accepted that the majority of HGSOC cases are probably derived from the serous tubal intraepithelial cancer that is subsequently implanted on the ovarian and/or pelvic surface. Low-grade serous ovarian cancers may originate from ovarian inclusion cysts derived either from the ovarian surface epithelium (the scenario is compliant with the classical theory) or from tubal epithelium. Endometrioid and clear cell ovarian cancers most probably originate from endometrial implants which arrive from the uterus due to so-called “retrograde menstruation” [8,9].

Histological types of ovarian cancer differ also by their molecular profile. HGSOC is characterized by *TP53* mutation prevalence and frequently shows loss of function of either of *BRCA* genes. In general, HGSOC has low mutational load, instead, showing a high copy number variation. Low-grade serous ovarian cancers typically have either *BRAF* or *KRAS* mutation. Endometrioid and clear cell cancers are characterized by microsatellite instability and mutations in *PIK3CA* and *PTEN*. In addition, mutation in the *ARID1A* gene is typical for clear cell cancer, while mutation in *CTNNB1* is frequent in endometrioid cancer. Mucinous cancers typically have *KRAS* mutation.

### 3.2. Uncertainty of Cellular Models of Ovarian Cancer

**Figure 14:**
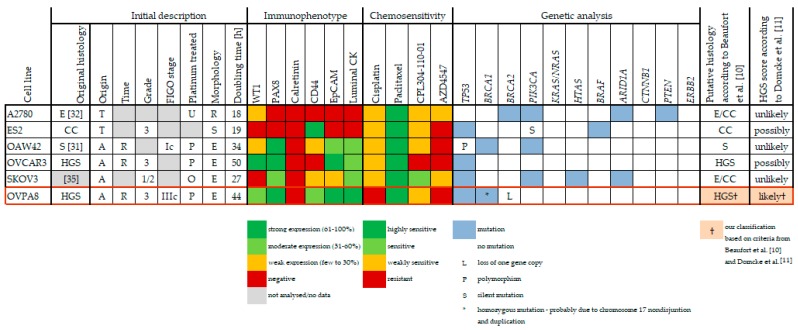
Summary of the OVPA8 cell line characteristics in comparison to five commercially available ovarian cancer cell lines. Original histology: CC—clear cell, E—endometrioid, HGS—high-grade serous, S—serous; Origin: A—ascitic fluid, T—tumor; Time: R—relapsed disease; Platinum treatment: U—untreated, P—platinum-based chemotherapy, O—other chemotherapy; Morphology: E—epithelial, R—round, S—spindle. Genetic data for commercially available cell lines were taken from [10] (except for *TP53* and *BRCA* genes).

In our study we used five commercially available ovarian cancer cell lines to compare with OVPA8: SKOV3, A2780, ES2, OAW42, and OVCAR3. The results of molecular and phenotypic characterization, as well as the cytotoxicity analysis for all these cell lines, are summarized in Figure 14.

Of the five control cell lines only OVCAR3 is undoubtedly of HGSOC origin, but all these lines are widely used in ovarian cancer research, particularly SKOV3 and A2780. The SKOV3 line is frequently cited as “serous’’, but it has been only vaguely reported in the original paper as “adenocarcinoma cell line derived from the ascitic fluid of ovarian cancer patient” [31]. The A2780 was originally described as a cell line established from an “ovarian endometrioid adenocarcinoma tumor” [32].

Domcke et al., who analyzed the data from The Cancer Cell Line Encyclopedia (CCLE) and compared it with those from The Cancer Genome Atlas, proposed a ranking of cell lines by their suitability as HGSOC models. In this ranking SKOV3 and A2780 are both classified as “unlikely HGSOC” [11]. The authors point out that these cells lack the major hallmarks of HGSOC, such as high copy number variation and mutations in *TP53* and *BRCA* genes. Instead, they have mutations in “non-HGSOC” genes: *ARID1A* (mutation typical for clear cell and endometrioid ovarian cancers) and *PI3K* (frequently found in clear cell ovarian cancers). Additionally, Anglesio et al., questioned the use of SKOV3 and A2780 as models of high-grade serous carcinoma [15], while Beaufort et al. have classified both of these lines as derived from “endometrioid or clear cell” ovarian cancers [10]. It was also shown that SKOV3 xenografts grown in nude mice have clear cell morphology with a massive accumulation of glycogen [33]. In summary, SKOV3 cells most probably represent clear cell ovarian cancer, although some uncertainties still exist due to putative *TP53* mutation in this cell line (see below). The A2780 seems to be derived from endometrioid ovarian cancer, as originally stated.

OVCAR3 is the third most prevalent ovarian cancer cell line. It was derived from the ascitic fluid from a patient with recurrent ovarian cancer diagnosed as poorly differentiated papillary adenocarcinoma [32]. OVCAR3 was later confirmed as HGSOC [10] or “possibly HGSOC” [11].

OAW42 and ES2 cell lines are less frequently used in the research, but their histological origin is also unclear. ES2 is distributed as a “clear cell carcinoma” line, however, its original histology has not been reported in the source reference [34]. Clear cell characteristics of ES2 is also proposed by Beaufort et al. [10]. On the contrary, Anglesio et al. have shown that ES2 xenografts in nude mice lack glycogen-containing clear cells [15], and in [11], the ES-2 line was classified as “possibly HGSOC” (Figure 14).

OAW42 was originally assigned as a “serous” cell line and is still regarded as such [10,35], but with some indications of “unlikely high-grade” histology [11] (Figure 14). On the other hand, some inconsistencies still remain, as OAW42 has mutations in *ARID1A* and *PIK3CA* genes, which are typical for endometrioid and clear cell cancers.

### 3.3. Stability of Cellular Models

The question of *TP53* mutation in SKOV3 cells is a good illustration of a wider problem of the stability of cellular model systems. Ikediobi et al. [16] were first to detect hom c.del267C frame-shift mutation in SKOV3 line, which we also detected by Sanger sequencing. Beaufort et al. [10] checked for *TP53* mutation in SKOV3 using two methods: tagged-amplicon deep sequencing and SOLiD exon sequencing and c.del267C was detected only by the first method. Elias et al. [36] reported deletion/frameshift mutation (further unspecified) in SKOV3-cis, a cisplatin resistant derivative of SKOV3 line. On the contrary, Anglesio et al. [15] reported “no change” in *TP53* sequence in SKOV3 cells and this information was repeated in Ince et al. [22]. Similarly, Domcke et al. [11] based their analyses on the data from CCLE which does not list *TP53* mutation in SKOV3.

In our opinion, these discrepancies may result from the fact that SKOV3 line has been in use since 1973 and many different clones could develop during propagation. Other possibility is that these inconsistencies result from different sequencing methods applied by different laboratories. This is just one example illustrating wide spectrum of possible differences in the model systems which are in use. Unfortunately, the same name not always denotes identical cell lines. 

Other example of such differences may be WT1 expression. Ince et al. [22] have observed no WT1 staining in A2780, while we have seen single WT1-positive cells in A2780. Differences were observed also in EpCAM expression in OVCAR3 cells. In [11] these cells were negative for EpCAM staining, while in our hands they showed ubiquitous and moderately intensive staining. Additionally, CD44 expression was discordant between our study and [10] for three cell lines: SKOV3, OVCAR3, and OAW42. 

Of note, in our study, five commercial ovarian cancer cell lines, SKOV3, A2780, ES2, OAW42, and OVCAR3, recalled morphological characteristics described in [10] (Figure 4), indicating that this feature is stable. Interestingly, Beaufort et al. [10] noted significant association between morphology (epithelial, spindle or round), and the origin: 14 out of 19 cell lines with epithelial morphology originated from ascites. Epithelial cell lines were more often of serous origin (83%), as compared to round (33%) and spindle (56%) cell lines. The morphological subtype was also significantly associated with prior platinum-based chemotherapy (10/14 epithelial had previously received platinum-based treatment versus all round and spindle lines which were derived from untreated patients) [10]. The OVPA8 cell line with its epithelial morphology fits into this model, being derived from platinum pre-treated, (high-grade) serous ovarian cancer and originating from ascites (Figure 14).

### 3.4. Cellular Models of HGSOC

Many biological drugs and new therapeutic approaches have been recently developed which improve survival of ovarian cancer patients [37,38]. To achieve further progress in ovarian cancer therapy we must better understand the biology of this disease. Apparently, for this purpose we need reliable in vitro models, especially for HGSOC. So far, numerous studies claiming to deal with HGSOC are performed with cell lines which do not correspond to this histological type. This is probably partially due to the lack of awareness of the problem and the shortage of well-defined and well-characterized models of HGSOC. In addition, some popular ovarian cancer cell lines have some practical advantages, i.e., they have low culture requirements, short doubling time, grow easily in vitro and in vivo. For these trivial reasons they may be preferentially chosen by researchers irrespective of their scientific utility.

Domcke et al. [11] suggested KURAMOCHI and OVSAHO cell lines as top HGSOC models based on their high genomic similarity to high-grade serous ovarian cancers from TCGA. These two lines together with JHOS4 (other top ranked candidate in [11]) were further studied by Elias et al. [36], who confirmed their HGSOC properties but discovered some performance limitations, e.g., poor growth in SCID mice (particularly observed for JHOS4).

Recently, Ince et al. developed Ovarian Carcinoma Modified Ince (OCMI) medium which favors efficient establishment of ovarian cancer cell lines from patients material. They developed 25 new ovarian cancer cell lines. Five of them have HGSOC origin and HGSOC features and two are described as “consistent with HGSOC”. These cell lines are available from the Sylvester Comprehensive Cancer Center Live Tumor Culture Core at the UM Miller School of Medicine and they are already in use [23,39], although not in the studies devoted to HGSOC, so far.

The OVPA8 is another cell line which will be available to the scientific community. The results of our detailed analyses confirm that the OVPA8 cells have morphologic, molecular, and functional features of HGSOC. We also believe that it has some practical advantages: it is resilient to adverse culture conditions (high confluency, old medium) and it is growing relatively fast (doubling time—44 h). It is already quite well characterized and further studies on its characterization are in progress. Thus, we consider that OVPA8 can become a valuable model for studies on high-grade serous ovarian cancer.

## 4. Materials and Methods

### 4.1. Establishment and Maintenance of the Cell Line from Malignant Ascites

A patient with relapsed ovarian cancer was treated at the Maria Skłodowska-Curie Institute—Oncology Center, Gliwice Branch. When she developed malignant ascites, she was qualified for paracentesis and IP chemotherapy. Ten liters of ascitic fluid were drained out and one liter was delivered to the research department (Center for Translational Research and Molecular Biology of Cancer), within scientific project no. IV.3.A.9 (2011). The project was accepted by the Bioethics Committee of the Maria Skłodowska-Curie Institute—Oncology Center, Gliwice Branch (KB/430-3618) on 23 February 2011. The patient gave informed consent for using the fluid for scientific puropses.

A starting amount of 1 L of ascitic fluid was used for the recovery of cancer cells (Figure 2) according to Langdon et al. [12]. The fluid was supplemented with heparin (10,000 U/L), transferred into two sterile 0.5 L bottles and centrifuged for 20 min at 3000× *g* in 4 °C. The supernatant was filtered, aliquoted, and stored at −20 °C. The cell pellet was suspended in PBS (phosphate-buffered saline 10 mL per 0.5 L bottle) and red blood cells were removed using centrifugation with Histopaque (Sigma-Aldrich, St. Louis, MO, USA). Briefly, the cell suspension (10 mL) was placed carefully onto Histopaque (10 mL) in 50 mL Falcon tubes and centrifuged at 2000× *g* for 30 min at room temperature. The mononuclear cells remaining at the interface were aspirated by Pasteur pipette and transferred to a new tube, then washed twice with 10 mL PBS and centrifuged at 1000× *g* for 10 min. At this point, the cells were suspended in the culture medium and counted. Aliquots of 10^6^ cells were cultured in 6 mL medium in 25 cm^2^ flasks. The first 10 passages of the cell culture were maintained in RPMI medium (Sigma-Aldrich) supplemented with sodium pyruvate, glucose, Na_2_HCO_3_, insulin (2.5 µg/mL; Sigma-Aldrich), penicillin (100 U/mL; Sigma-Aldrich), streptomycin (0.1 mg/mL; Sigma-Aldrich), amphotericin B (0.25 µg/mL; Sigma-Aldrich), l-glutamine (2 mM; Sigma-Aldrich), and 10% autologous ascitic filtrate. Subsequently, the ascitic filtrate was substituted with 10% fetal bovine serum (FBS; Gibco, Waltham, MA, USA) and insulin was no longer added.

In order to remove fibroblasts and mesothelial cells from the initial culture, we applied selective trypsinization. Both fibroblasts and mesothelial cells tend to detach rapidly from plastic after short-term trypsinization. Treatment with trypsin for 2 min was used to detach these cells from the culture plate without removing epithelial cancer cells (Figure 3). Detached cells were discarded and fresh medium with FBS was immediately added to inactivate the remaining trypsin.

### 4.2. Cell Lines and Culture Conditions

ES2, OVCAR3, and SKOV3 cell lines were purchased from the American Type Culture Collection (ATCC, Manassas, VA, USA). A2780 and OAW42 lines were purchased from the European Collection of Cell Culture (ECACC, Porton Down, UK). All established cell lines (except of SKOV3) and the new cell line OVPA8 were grown in RPMI medium (Roswell Park Memorial Institute medium) (Sigma-Aldrich) supplemented with 5% (A2780), 10% (ES2, OAW42, OVPA8) and 20% (OVCAR3) fetal bovine serum (FBS; Gibco) and 1% penicillin-streptomycin-amphotericin B solution (Sigma-Aldrich). Additional supplements were used for culturing OAW42 (10 mg/mL insulin; Sigma-Aldrich) and OVCAR3 (0.01 mg/L insulin, glucose—4.5 g/L). SKOV3 cells were cultured in McCoy’s 5A (Sigma-Aldrich) supplemented with 10% FBS.

### 4.3. Detection of Virus Contamination

RNA and DNA were isolated with a GeneJet Viral DNA and RNA Purification Kit (Thermo Fisher Scientific, Waltham, MA, USA). cDNA synthesis was conducted using Maxima H Minus First Strand cDNA Synthesis Kit (Thermo Fisher Scientific). EBV detection was performed using an EBV Real-TM Quant Kit (Sacace Biotechnologies, Como, Italy). All procedures were performed according to manufacturers’ protocols. Detection of HIV, HCV, and HBV was performed using primers published by Uphoff et al. [40] and experimentally-optimized PCR conditions (Table 3). *B2M* (β-2-Microglobulin) and *BNIP3* (BCL2 Interacting Protein 3) specific primers were used as internal controls. The PCR products were identified by agarose gel electrophoresis and visualized by ethidium bromide staining.

### 4.4. Detection of Mycoplasma Contamination

Half a milliliter of medium from 2–3 day old cell culture was transferred into a 1.5 mL tube and centrifuged at 600× *g* for 2 min to eliminate cellular debris. The supernatant was transferred to the new tube and centrifuged at 16,100× *g* for 15 min. The pellet was suspended in water and heated at 95 °C for 10 min. Detection of mycoplasma DNA was done by PCR, using appropriate primers and experimentally-set PCR conditions (Table 3). 18S rRNA-specific primers were used to control the reaction efficiency (internal control). The PCR products were identified by agarose gel electrophoresis and ethidium bromide staining. All cell cultures were regularly tested for mycoplasma contamination.

### 4.5. Cell’s Morphology and Growth Characteristics

For morphological studies of the new OVPA8 cell line and the five established ovarian cancer cell lines, the cells were grown in 25 cm^2^ culture flasks and observed daily using phase contrast microscopy to assess cell shape, size, and growth pattern.

Population doubling time (DT) of the OVPA8 cell line was determined in a hemocytometer chamber. Briefly, 2.5 × 10^5^ cells were plated in a 25 cm^2^ flask. Viable cells were counted at 120 h (five days) after seeding. Culture media were changed every three days. The average number of cells was calculated in six different experiments. To calculate the population doubling time, we used the following formula: DT = *T* ln2/ln(*X*e/*X*b), where *T* is the duration of culture (h), *X*e is the cell number at the end of the incubation time, and *X*b is the cell number at the beginning of the incubation time.

Plating efficiency was estimated by comparing the ability of OVPA8 and the commercial ovarian cancer cell lines to form colonies. Cells were seeded at 1.5–3.0 × 10^3^ cells/6-cm polystyrene plates and incubated until the formation of colonies (8–14 days, depending on the cell line). The culture medium was changed once a week. Colonies were fixed with methanol/formalin 1:1 (*v*/*v*), stained with 0.5% crystal violet for 15 min at 37 °C, and documented using a G:Box Imaging System and GeneTools software (Syngene, Bangalore, India).

For cell migration assay cells were harvested, resuspended in the serum-free medium, and seeded at a concentration of 7.5 × 10^4^ cells/well (300 µL/insert) on the upper surface of a 24-well transwell insert with a pore size of 8 μm (Falcon^®^ Corning, New York, NY, USA), pre-treated with fibronectin (10 µg/mL; Merck KGaA, Darmstadt, Germany). The insert was placed in a 24-well plate that contained fresh medium with 10% FBS (800 µL/well). Plates were incubated at 37 °C in a 5% CO_2_ incubator for 4, 24, and 48 h. Afterwards, the wells were disassembled and the cells remaining at the inner side of the membrane were removed with a cotton swab, while the cells present on the outer side of the membrane were fixed by drying overnight at room temperature. The next day, the cells were stained with crystal violet, then the stain was released using 100 µL of 10% acetic acid and the absorbance of the solution was measured on a Synergy 2 multi-mode plate-reader (BioTek Instruments, Winooski, VT, USA) at a wavelength of 595 nm. The mean from three measurements of three membranes per cell line was calculated.

A matrigel cell invasion assay was performed using a 24-well plate with transwell inserts (pore size of 8 μm; Falcon^®^ Corning), pre-treated with fibronectin (10 µg/mL; Merck KGaA) and coated with matrigel (200 µg/mL, 100 µL/insert; Corning). 7.5 × 10^4^ cells suspended in 200 µL medium without serum were seeded in the upper chamber, whereas the lower chamber was filled with medium containing 10% FBS (800 µL/well). The whole plate was incubated at 37 °C in a 5% CO_2_ incubator for 96 h to allow cell invasion. Then, the tumor cell invasiveness was determined as previously described in the cell migration assay. The mean from three measurements of three membranes per cell line was calculated.

### 4.6. Growth Curves and Chemosensitivity Assay

Cells were seeded in 96-well plates in pentaplicate. After 24 h cells were treated with different concentrations of cisplatin (0.1–20 µM; Ebewe Pharma, Unterach, Austria), paclitaxel (0.00156–30 µM; Ebewe Pharma), and FGFR inhibitors: AZD454 (0.0001–10 µM; Astra Zeneca, London, UK) or CPL-304-110 (0.0001–10 µM; Celon Pharma). After a three-day treatment with various concentrations of the compound the cell viability was assessed using AlamarBlue assay. GraphPad Prism software was used to fit dose response curves and calculate the half maximal inhibitory concentration values (IC_50_) with error and 95% confidence intervals.

### 4.7. Short Tandem Repeat (STR) Analysis

The AmpFLSTR NGM PCR Amplification Kit (Applied Biosystems) was used according to the manufacturer’s protocol and reaction products were analyzed using a Genetic Analyser 3500 (Applied Biosystems) unit to generate an STR fingerprint. Data analysis was carried out with the GeneMapper 5 software (Thermo Fisher Scientific).

### 4.8. Immunocytochemical Staining (ICC)

ICC was performed using antibodies against the following proteins: WT1 (clone 6F-H2; Dako, Santa Clara, CA, USA), PAX8 (clone MRQ-50; CellMarque, Darmstadt, Germany), calretinin (colne DAK-Calret1; Dako), luminal cytokeratin 19 (CK19, clone A53-B2.26; Selmark), CD44 (clone DF1485; Dako), CD68 (clone PG-M1; Dako), EpCAM (clone BerEP4; Dako), FN1 (Dako), and POSTN (Abcam). Reactions were performed on cytospined cell preparations of five established ovarian cancer lines and early and late passages of OVPA8 cultures.

Detection systems were used as follows: ImmPRESS™ REAGENT Anti-Rabbit Ig (Vector, Burlingame, CA, USA) for FN1 and POSTN, and EnVisionTM FLEX+, Mouse, High pH, and (Link) System (Dako) for other proteins. FN1 and POSTN were additionally detected using diaminobenzidine (DAB Peroxidase Substrate Kit, Vector) substrate.

For microscopic evaluation (Axiophot Microscope; Zeiss, Oberkochen, Germany), the preparations were counterstained with hematoxylin and mounted with Dako Ultramount Aqueous Permanent Mounting Medium (Dako). Pictures were acquired using a Pannoramic 250 Flash II scanner (3DHISTECH). The intensity of staining was evaluated as follows: − = negative; + = weak (few to 30% of cells); ++ = moderate (31–60%); +++ = strong (61–100%).

### 4.9. FACS Analysis

Surface expression of CD44 and CD133 markers was detected using flow cytometry (FC). The cells were harvested and stained with fluorochrome-conjugated antibodies: anti-CD44-FITC (BD Biosciences, San Jose, CA, USA) and anti-CD133-PE (Miltenyi Biotec, Bergisch Gladbach, Germany). Protein expression was measured for a minimum of 10,000 events using BD FACS Canto III (BD Biosciences). Detection of a side population was done with Hoechst 33,342 dye (Sigma-Aldrich) by BD FACS Aria III (BD Biosciences).

### 4.10. Karyotype Analysis

G-banding was performed according to classical protocol for adherent cells [41]. Briefly, OVPA8 cell cultures in their logarithmic growth phase were exposed for 45 min. to Colcemid solution (Invitrogen) at final concentration of 0.1 µg/mL. Afterwards, the cells were treated with pre-warmed hypotonic lysis solution (0.075 M KCl) at 37 °C for 10 min and fixed with Carnoy’s solution (3:1 methanol:glacial acetic acid) at room temperature. Cell suspension was dropped onto glass slides, air-dried and treated by pre-warmed 0.25% trypsin solution at 37 °C for 3–10 s. Staining was done using 4% Giemsa solution in Sorensen buffer for 10 min. Slides were rinsed in water and air-dried at room temperature. Cytogenic analysis was done using Ikaros software (MetaSystems, Heidelberg, Germany). Seven metaphase spreads were analyzed and chromosomes were classified according to the International System for Human Cytogenetic Nomenclature (ISCN).

### 4.11. Mutation Testing

Genomic DNA was isolated using Genomic mini AX Body Fluids Kit (A&A Biotechnology, Gdynia, Poland). Three most frequent *BRCA1* mutations occurring in Polish population: C61G (c.181T>G; p.Cys61Gly), 4153delA (c.4035delA; p.Glu1346Lysfs) and 5382insC (c.5266dupC; p.Gln1756Profs) were checked in OVPA8 and five other cell lines with allele-specific oligonucleotide polymerase chain reaction (ASO-PCR) (Table 4) according to Brozek et al. [42].

Next-generation sequencing (NGS) was performed to search for other mutations in *BRCA1* and *BRCA2* genes in OVPA8 cells. Automated library construction was performed with Oncomine *BRCA* Research Assay, Chef-Ready on an Ion Chef instrument (Thermo Fisher Scientific). Pooled, equalized, and diluted to 100 pM libraries were used for automated NGS template preparation on an Ion Chef instrument (Thermo Fisher Scientific) and subsequent sequencing using Ion Torrent Personal Genome Machine (PGM) system and the Ion 316™ Chip Kit v2 BC (Thermo Fisher Scientific), according to manufacturer’s protocols. The data was analyzed using Ion Reporter Software (Thermo Fisher Scientific) and ANNOVAR software was used to annotate genetic variants. Somatic gene loss events were detected using Ion Reporter’s built-in algorithm for CNV analysis which performs a statistical comparison of the distribution of normalized amplicon coverage values across the entire set of *BRCA1* and *BRCA2* coding exons.

The Ion AmplSeq™ Library Kit 2.0 (Thermo Fisher Scientific) and the Ion AmpliSeq Cancer Hotspot Panel v2 (Thermo Fisher Scientific) was used to prepare the library for analysis of approximately 2800 COSMIC mutations in 50 cancer-related genes (*ABL1*, *EGFR*, *GNAS*, *KRAS*, *PTPN11*, *AKT1*, *ERBB2*, *GNAQ*, *MET*, *RB1*, *ALK*, *ERBB4*, *HNF1A*, *MLH1*, *RET*, *APC*, *EZH2*, *HRAS*, *MPL*, *SMAD4*, *ATM*, *FBXW7*, *IDH1*, *NOTCH1*, *SMARCB1*, *BRAF*, *FGFR1*, *JAK2*, *NPM1*, *SMO*, *CDH1*, *FGFR2*, *JAK3*, *NRAS*, *SRC*, *CDKN2A*, *FGFR3*, *IDH2*, *PDGFRA*, *STK11*, *CSF1R*, *FLT3*, *KDR*, *PIK3CA*, *TP53*, *CTNNB1*, *GNA11*, *KIT*, *PTEN*, *VHL*). NGS was performed as described above, with the exception that 45 pM of library was taken for template preparation.

*TP53* mutations in exons 5–9 were analyzed in OVPA8 and five other cell lines by Sanger sequencing (Table 4), according to Krześniak et al. [43].

Fluorescence in situ hybridization (FISH) was performed to visualize chromosome 17 and *TP53* locus. OVPA8 cells were fixed on microscope slides and stained using SpectrumGreen-labeled CEP17 and SpectrumOrange-labeled 17p13.1 (*TP53*) probes (Vysis TP53/CEP17 FISH Probe Kit, Abbott, Lake Bluff, IL, USA). Both interphase and metaphase images were analyzed using an AxioImager.M2 (Zeiss) fluorescence microscope and AxioVision Microscope software (Zeiss).

### 4.12. Semi-Quantitative Reverse-Transcription PCR (RT-PCR)

Total RNA was isolated with RNeasy Mini Kit (Qiagen, Venlo, Netherlands). RNA concentration and purity was checked with a NanoDrop spectrophotometer (Thermo Fisher Scientific). cDNA was synthesized with a Maxima H Minus First Stand cDNA Synthesis Kit (Thermo Fisfer Scientific) according to the manufacturer’s suggestions. Semi-quantitative PCR for fibronectin 1 (*FN1*) and periostin (*POSTN*) was conducted using appropriate primers and an AmpliTaq Gold Polymerase Kit (Thermo Fisher Scientific) in experimentally-adjusted temperature conditions (Table 5). 18S rRNA was amplified as an internal standard to control for PCR efficiency. The PCR products were separated by electrophoresis in a 2% agarose gel containing ethidium bromide (0.5 µg/mL).

### 4.13. Western Blot Analysis

Approximately 10^7^ cells were lysed with Radio-ImmunoPrecipitation Analysis (RIPA) buffer (Thermo Fisher Scientific) for 10 min on ice, and then centrifuged at 10,000× *g* at 4 °C to remove cell debris. A total of 50 µg of cellular protein extracts were separated using 8% SDS-PAGE under reducing conditions and transferred onto polyvinylidene difluoride (PVDF) membranes (Serva, Heidelberg, Germany). To detect the proteins of interest, anti-FN1 (Dako) and anti-POSTN (Abcam) antibodies were used. To control for protein loading uniformity anti-HSC70 antibody (Acris Antibodies, Herford, Germany) was used. Visualization was performed with secondary antibodies coupled to horseradish peroxidase (Vector) and Immobilon Western Chemiluminescent HRP Substrate (Millipore, Darmstadt, Germany).

### 4.14. Statistical Analysis

Unless otherwise stated, all data were shown as mean ± standard deviation (SD) of the mean. Univariate statistical significance was determined by one-way analysis of variance (ANOVA) with Bonferroni’s correction for pairwise comparisons. Difference significance between two groups was determined by T test for independent samples. A *p*-value of less than 0.05 was considered statistically significant. STATISTICA 12 (StatSoft, Tulsa, OK, USA) was used for the statistical analyses. 

## Figures and Tables

**Table 1:** Short tandem repeats (STR) profile of OVPA8 cell line established using an AmpFLSTR NGM PCR Amplification Kit (Applied Biosystems, Waltham, MA, USA). Question mark (?) was inserted there because the reading in this position was ambigous.

Loci	Results
AMELO (amelogenin)	X
D10S1248	14, 15
vWA (40th intron of von Willebrand Factor gene)	14
D16S539	12, 14
D2S1338	21, 25
D8S1179	11, 14
D21S11	29, 30
D18S51	12
D22S1045	15
D19S43	13, 15
THO1 (1st intron of tyrosine hydroylase gene)	8
FGA (3rd intron of alpha fibrinogen gene)	22, 25
D2S441	?, 14
D3S1358	15
D1S1656	15
D12S391	21

**Table 2:** Drug sensitivity of OVPA8 and other ovarian cancer cell lines. Cytotoxicity of cisplatin, paclitaxel and two different FGFR inhibitors was tested and IC50 values were calculated using GraphPad Prism software (GraphPad).

Cell Line	Agent	IC50 (µM)
OVPA8	Cisplatin	16.27
	Paclitaxel	0.001
	CPL304-110-01	3.05
	AZD4547	>10
SKOV3	Cisplatin	3.17
	Paclitaxel	0.0055
	CPL304-110-01	0.99
	AZD4547	7.67
OVCAR3	Cisplatin	1.48
	Paclitaxel	<0.0016
	CPL304-110-01	>10.00
	AZD4547	>10.00
OAW42	Cisplatin	2.78
	Paclitaxel	0.0033
	CPL304-110-01	3.51
	AZD4547	>10.00
ES2	Cisplatin	1.63
	Paclitaxel	<0.0016
	CPL304-110-01	2.32
	AZD4547	>10
A2780	Cisplatin	1.03
	Paclitaxel	0.004
	CPL304-110-01	1.65
	AZD4547	9.73

**Table 3:** Primers and conditions used for viruses and mycoplasma detection by PCR; F—forward primer, R—reverse primer.

Target	Sequences of Primers (5′→3′)	Amplicon Size	Annealing Temperature
HBV (hepatitis B virus)	F: 5′-AAGCTGTGCCTTGGGTGGCTTTG-3′	570 bp	58 °C
	R: 5′-CGAGATTGAGATCTTCTGCGACG-3′		
HCV (hepatitis C virus)	F: 5′-GCCATGGCGTTAGTATGAGTGTC-3′	259 bp	58 °C
	R: 5′-ATGCACGGTCTACGAGACCTCC-3′		
HIV (human immunodeficiency virus)	F: 5′-ATAATCCACCTATCCCAGTAGGAGAAAT-3′	116 bp	58 °C
	R: 5′-TTTGGTCCTTGTCTTATGTCCAGAATGC-3′		
B2M (β-2-microglobulin)	F: 5′-CTGGGTTTCATCCATCCGACA-3′	212 bp	59 °C
	R: 5′-GTCTCGATCCCACTTAACTATCTTGG-3′		
BNIP3 (BCL2-interacting protein 3)	F: 5′-CGGATTGGGGATCTATATTGGAAG- 3′	187 bp	59 °C
	R: 5′-AGGAACGCAGCATTTACAGAACAA-3′		
Mycoplasma	F: 5′-GGCGAATGGGTGAGTAACACG-3′	500 bp	55 °C
	R: 5′-CGGATAACGCTTGCGACCTAT-3′		
18S rRNA (18S ribosomal RNA)	F: 5′-CATGGCCGTTCTTAGTTGGTG-3′	193 bp	55 °C
	R: 5′-GTGCAGCCCCGGACATCTAA-3′		

**Table 4:** Primers and conditions used for analysis of mutations in *BRCA1* and *TP53* genes; F—forward primer, R—reverse primer.

Target	Sequences of Primers (5′→3′)	Annealing Temperature
*BRCA1*: C61G (c.181T>G; p.Cys61Gly)	F: 5′-CTCTTAAGGGCAGTTGTGAG-3′	65 °C
	R: 5′-TTCCTACTGTGGTTGCTTCC-3′	
*BRCA1*: 4153delA (c.4035delA; p.Glu1346Lysfs)	F: 5′-TATTGGCAAAGGCATCTCAG-3′	65 °C
	R: 5′-GCCAAAGATGACGTCCTAGC-3′	
	DEL: 5′-GGAATTGGTTTCAGATGATGAG-3′	
*BRCA1*: 5382insC (c.5266dupC; p.Gln1756Profs)	F: 5′-ATATGACGTGTCTGCTCCAC-3′	65 °C
	RINS: 5′-CCTTTCTGTCCTGGGGATT-3′	
	R: 5′-GGGAATCCAAATTACACAGC-3′	
*TP53* (exones 5–6)	F: 5′-GTTGCAGGAGGTGCTTACA-3′	61 °C
	R: 5′-GAGGTCAAATAAGCAGCAGG-3′	
*TP53* (exones 7–9)	F: 5′-GAGCGAGATTCCATCTCAA-3′	53 °C
	R: 5′-CAGTGCTAGGAAAGAGGCAA-3′	

**Table 5:** Primer sequences and conditions used for semi-quantitative PCR analysis of *FN1* and *POSTN* expression; F—forward primer, R—reverse primer.

Target	Sequences of Primers (5′→3′)	Amplicon Size	Annealing Temperature
*FN1*	F: 5′-CAACTCTGTCAACGAAGGCTTG-3′	493 bp	59 °C
	R: 5′-CTGAGAATACTGGTTGTAGGACTGG-3′		
*POSTN*	F: 5′-CTATCCAGCAGACACACCTGTTG-3′	347 bp	51 °C
	R: 5′-TTTCCACAGGCACTCCATCAAT-3′		
18S rRNA	F: 5′-CATGGCCGTTCTTAGTTGGTG-3′	193 bp	55 °C
	R: 5′-GTGCAGCCCCGGACATCTAA-3′		

## Supplementary Materials

Supplementary materials can be found at http://www.mdpi.com/1422-0067/19/7/2080/s1.

## Abbreviations

ATCC	American Type Culture Collection
B2M	β-2-Microglobulin
BSL	Biosafety level
BNIP3	BCL2-interacting protein 3
CCLE	Cancer cell line encyclopedia
CC	Clear cell (ovarian cancer)
CGH	Comparative genome hybridization
CK19	Luminal cytokeratin 19
CT	Computed tomography
DT	Doubling time
EBV	Epstein-Barr virus
ECACC	European Collection of Authenticated Cell Cultures
E	Endometroid (ovarian cancer)
EpCAM	Epithelial cell adhesion molecule
FACS	Fluorescence-activated cell sorting
FGFR	Fibroblast growth factor receptor
FISH	Fluorescence in situ hybridization
FN1	Fibronectin 1
HBME-1	Hector Battifora mesothelial epitope 1
HBV	Hepatitis B virus
HCV	Hepatitis C virus
HE	Hematoxylin-eosin staining
HGSOC	High-grade serous ovarian cancer
HIV	Human immunodeficiency virus
ICC	Immunocytochemistry
IP	Intraperitoneal (chemotherapy)
ISCN	International System for Human Cytogenetic Nomenclature
IV	Intravenous (chemotherapy)
LGSOC	Low-grade serous ovarian carcinoma
NGS	New generation sequencing
OS	Overall survival
OSE	Ovarian surface epithelium
PAX8	Paired Box 8
POSTN	Periostin
PVDF	Polyvinylidene difluoride
RT-PCR	Reverse-transcription polymerase chain reaction
SD	Standard deviation
SDS-PAGE	Sodium dodecyl sulfate–polyacrylamide gel electrophoresis
SNP	Single nucleotide polymorphism
SP	Side population
STR	Short tandem repeat
